# 

**Published:** 1828-10

**Authors:** 


### MONTHLY LIST OF MEDICAL BOOKS.

[*Medical Works cannot be entered on this List except a copy be sent for the purpose; the titles of Books having frequently been transmitted to us, as published, which have not appeared for weeks, or even months, after.*]

Medical Essays on Fever, Inflammation, Rheumatism, Disease of the Heart, &c. By JOSEPH BROWN, M.D. &c.

A Letter, addressed to his Excellency the Right Honourable General the Earl of Chatham, K.G. Governor of Gibraltar, &c. &c. &c. relative to the Febrile Distempers of that Garrison. By W. W. FRASER, Esq.

Remarks on the Treatment of the Insane, and the Management of Lunatic Asylums; being the Substance of a Return from the Lincoln Lunatic Asylum to the Circular of his Majesty's Secretary of State; with a Plan. By E. P. CHARLESWORTH M.D.

A Manual of Modern Surgery, founded upon the Principles and Practice lately taught by Sir ASTLEY COOPER, Bart. F.R.S. and JOSEPH HENRY GREEN, F.R.S. Edited by THOMAS CASTLE, F.L.S. &c.

Elements of Descriptive and Practical Anatomy, for the use of Students. By JONES QUAIN, A.B. M.B. Member of the Royal College of Surgeons, and one of the Lecturers on Anatomy in the Medical School, Aldersgate-street.

### NOTICES.

J. M. *is informed that Medical Degrees are granted in the University of St. Andrews under certain regulations.*

